# Transcranial Direct Current Stimulation Augments Perceptual Sensitivity and 24-Hour Retention in a Complex Threat Detection Task

**DOI:** 10.1371/journal.pone.0034993

**Published:** 2012-04-12

**Authors:** Brian Falcone, Brian A. Coffman, Vincent P. Clark, Raja Parasuraman

**Affiliations:** 1 Center of Excellence in Neuroergonomics, Technology, and Cognition (CENTEC), George Mason University, Fairfax, Virginia, United States of America; 2 The Mind Research Network and Lovelace Biomedical and Environmental Research Institute, University of New Mexico, Albuquerque, New Mexico, United States of America; Royal Holloway, University of London, United Kingdom

## Abstract

We have previously shown that transcranial direct current stimulation (tDCS) improved performance of a complex visual perceptual learning task (Clark et al. 2012). However, it is not known whether tDCS can enhance perceptual sensitivity independently of non-specific, arousal-linked changes in response bias, nor whether any such sensitivity benefit can be retained over time. We examined the influence of stimulation of the right inferior frontal cortex using tDCS on perceptual learning and retention in 37 healthy participants, using signal detection theory to distinguish effects on perceptual sensitivity (*d′*) from response bias (*ß*). Anodal stimulation with 2 mA increased *d′*, compared to a 0.1 mA sham stimulation control, with no effect on *ß*. On completion of training, participants in the active stimulation group had more than double the perceptual sensitivity of the control group. Furthermore, the performance enhancement was maintained for 24 hours. The results show that tDCS augments both skill acquisition and retention in a complex detection task and that the benefits are rooted in an improvement in sensitivity (*d′*), rather than changes in response bias (*ß*). Stimulation-driven acceleration of learning and its retention over 24 hours may result from increased activation of prefrontal cortical regions that provide top-down attentional control signals to object recognition areas.

## Introduction

Perceptual sensitivity, as measured by the signal detection theory metric *d′* [1] and related indexes [2], is a basic measure of perceptual capability. It has long been used to assess perceptual performance and learning [3]. For example, perceptual sensitivity measures can be used to evaluate acquisition of the ability to detect obscured or concealed objects of the type encountered in naturalistic scenes. This is an important skill that typically develops only after extensive training [4]. Examples include radiologists identifying tumors in MRI scans or security officers examining surveillance videos of people for suspects. Perceptual sensitivity is also diminished in many sensory disorders, as in hearing-impaired individuals or those with low vision [5]. Reduced perceptual sensitivity can also contribute to functional deficits in brain disorders such as head injury [6], schizophrenia [7], and Alzheimer's disease [8].

Identifying methods that can increase perceptual sensitivity in both healthy and clinical populations can have significant applications for clinical assessment, training, and research. Unfortunately, few methods exist to enhance perceptual sensitivity reliably and consistently. Many techniques only serve to alter participants' response bias, so that correct target detections (hits) may increase but at the cost of more false alarms, without a change in sensitivity [9]. Stimulant drugs such as amphetamine [10] and physostigmine [11] can increase sensitivity, but possess significant drawbacks, such as reduced effectiveness due to tolerance, the potential for addiction, and ethical issues raised by the use of pharmacological agents in healthy adults [12].

A newly emerging alternative is to use non-invasive brain stimulation to modulate neuronal activity, in particular transcranial direct current stimulation (tDCS). A number of tDCS studies have shown that it is possible to enhance human performance through the application of low-level DC current to the scalp while participants are engaged in simple perceptual, cognitive, and motor tasks. Examples include studies of motion discrimination [13], visual attention [14], working memory [15], and exploratory behavior [16]. For a recent review of these and other tDCS studies, see Ref [17]. However, it is unclear whether tDCS can reliably enhance perceptual sensitivity in detection tasks, particularly those involving complex targets and naturalistic scenes. Moreover, the duration of this sensitivity benefit is unknown. For tDCS to be a viable training technique, it would be desirable if its effects can be retained for hours, if not days.

In the present study we examined both of these issues by applying tDCS to scalp regions overlying the inferior frontal cortex of participants learning to perform a complex threat detection task. We hypothesized that tDCS would improve encoding of stimulus features during training and thereby accelerate learning. Signal detection theory analysis was used to examine effects of brain stimulation on perceptual sensitivity independently of response bias. We also investigated whether the effect of tDCS on sensitivity, if found, would be retained over a 24-hour period.

tDCS uses small DC electric currents (typically 1 to 2 mA) that are applied to the scalp. The technique is considered to be safe for experimental use in healthy subjects for up to about 30 minutes of stimulation [18]. The mechanism by which tDCS influences brain function is not precisely known, but is thought to involve alteration of the electrical environment of cortical neurons, specifically small changes in the resting membrane potential of neurons, so that they fire more readily to input from other neurons [19]. In vitro studies have shown that DC stimulation of rat hippocampal slices at low current levels decreases the threshold for neuronal firing [20]. We have also shown, using magnetic resonance spectroscopy in humans, that tDCS results in increased levels of glutamate, glutamine, and N-Acetylaspartic acid that remain elevated after current is turned off [21]. A positive (anodal) polarity is typically used to stimulate neuronal function and enhance behavioral performance. Conversely, a negative (cathodal) polarity is used to inhibit neuronal activity, although this has also been found to result in behavioral improvements under certain conditions [22].

Neuroimaging studies can help identify the key brain networks that are associated with the performance of a perceptual detection task and thereby help locate the scalp targets for anodal tDCS application. We used this approach in a functional magnetic resonance imaging (fMRI) study of a complex perceptual learning task requiring participants to identify concealed and camouflaged objects representing threats in a simulation of naturalistic warzone environments [23]. The task was modeled on the “DARWARS Ambush” virtual reality environment [24], which has been used to familiarize and train personnel prior to deployment to areas of military conflict. The objects that participants had to detect included bombs that were concealed by or disguised to look like everyday objects. Other threats involved people who were either enemy combatants in concealed locations or dressed to look like ordinary civilians, with subtle clues as to their identity. Participants performed the object detection task without feedback while undergoing fMRI scanning. Subsequently, they underwent training sessions with feedback outside of the MRI scanner on sequential days. MRI scans were repeated when they reached an intermediate level of performance and again when they attained expert level performance. Activation of brain regions associated with scenes containing concealed objects was compared to that for scenes without such targets at novice, intermediate, and expert stages of performance. Based on these findings, as well as on a Bayesian network analysis of the activated brain regions, the right inferior frontal cortex and the right parietal cortex were identified as areas with significant activation associated with performance and learning of the threat detection task and which are accessible using tDCS on the scalp. In a separate group of participants, anodal 2.0 mA tDCS was applied to the scalp areas overlying these regions. Task performance was significantly enhanced compared to a group receiving 0.1 mA tDCS (“sham” stimulation control group).

These results indicate that tDCS might provide an effective technique for efficient training of high-performance perceptual and cognitive skills in complex tasks. However, additional questions must be addressed before a firm conclusion can be reached on the training potential of tDCS. One issue that needs further examination in tDCS studies, especially those involving perceptual detection tasks, is whether brain stimulation enhances perceptual sensitivity as opposed to making participants more liberal or conservative in responding. The latter could result from non-specific changes in arousal, which can influence response bias [25]. For example, if tDCS only shifts response bias in a liberal direction so that participants are more likely to respond positively in a detection task, the hit rate will increase, even though there may be no change in the participant's ability to detect the target. Clark et al. [23] reported that tDCS increased the rate of correct responses in a threat detection task. However, a change in response bias could also lead to a larger rate of correct responses and cannot be distinguished from a change in perceptual sensitivity using a measure of correct response rates alone [1]–[3].

A second important issue is the degree of retention of performance benefits. That is, how long does the benefit of tDCS last? If performance improvement only lasts as long as stimulation or for a short time after, it would not be useful for producing long-term improvements in perceptual sensitivity. Accordingly, in the present study we examined whether a tDCS-related performance benefit in a complex threat detection task would, if obtained, be retained over a 24-hour period. A positive finding would then represent a starting point for exploring retention over longer periods of time and examination of other issues, such as transfer of training.

In summary, we used signal detection theory to examine whether tDCS applied to the scalp over right inferior cortex during acquisition of a complex threat detection task affects perceptual sensitivity (*d′*) as opposed to response bias (*ß*). We also investigated whether there would be retention of any performance benefit over a 24-hour period. We hypothesized that participants receiving 2.0 mA tDCS would show an increase in *d*′ but not in *β*, relative to participants receiving 0.1 mA, and that this effect would be significant immediately after training and again 24 hours later.

## Materials and Methods

### Ethics Statement

All human participants provided written informed consent to take part in the study, which was approved by the George Mason University Institutional Review Board.

### Participants

Participants were 37 adults (21 males, 16 females) aged 18–25 years (mean = 20.1 years). Prior to enrollment in the study, participants were screened and excluded for having a primary language other than English, a history of head injuries or concussions, left-handedness, current or previous history of mental, neurological, alcohol or drug abuse disorders, current prescription medication affecting central nervous system function, or uncorrected hearing or visual impairments. The participants were randomly assigned to one of two groups, an active stimulation group (N = 19) and a “sham” stimulation control group (N = 18).

### Threat Detection Task

**Figure 1:**
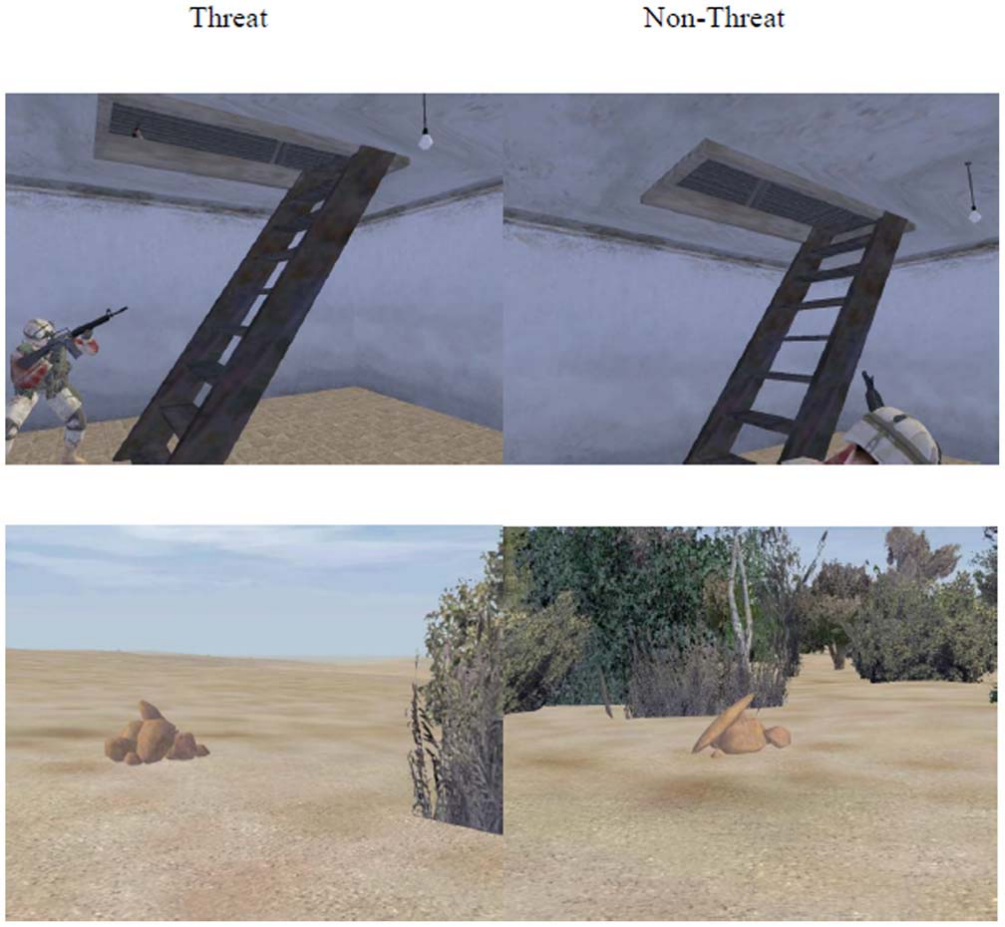
Examples of the concealed threat in images used for both test and training blocks. Similar scenarios could be repeated throughout the experiment but the presence of a threat varied from trial to trial. Top row, left image: an example of a concealed enemy combatant scenario, indicated by the barely visible tip of a firearm in the room at the top of the ladder. No threat is present in the right image. Bottom row, left image: example of a bomb that has been concealed by a stack of rocks. The bomb is indicated by a tiny object that is barely visible through the space between the rocks. No threat is present in the right image.

Short movies showing naturalistic scenes containing objects and people as well as still images extracted from those movies were taken from the “DARWARS Ambush” virtual reality software for presentation to participants [23], [24]. Participants were only told that they were to determine whether or not there was a threat present in the image, without being provided specific details as to what types of possible threats were present. Half of the scenes included specific concealed objects that indicated possible threats that participants had to detect, while the other half did not contain concealed objects. Examples of images with and without objects indicating possible threats are shown in Figure 1. Target objects that signified threats included concealed objects such as bombs that were hidden by, or disguised as, trash, deceased animals, fruit or other objects such as oil barrels, boxes, cars, toys. Bombs could also be indicated by trip wire or appear as a conspicuous unattended package. People could also signify threats and included enemy combatants such as snipers in various concealed locations, plainly-clothed suicide bombers, plainly-clothed individuals carrying a concealed weapon, or non-military personnel in conspicuous locations (e.g. sneaking up behind military personnel). In each case, similar scenes without target objects were created that differed by discernable characteristics indicating threat presence. The concealed target objects were subtle enough to be missed on first viewing but could be more readily identified after training. Therefore, detection accuracy was expected to be at chance levels during the initial phases of task performance. A discovery-learning paradigm was used in which participants were only told that they were to determine whether or not there was a threat present in the image, without being provided specific details as to what types of possible threats were present. With experience and interaction with the task during training, however, participants could learn what to look for in the images.

During the training blocks, still images were presented for 2 s each, followed by a 1 s inter-stimulus interval (ISI) consisting of a blank screen with a crosshair fixation. Participants were required to make a button press within 3 s of stimulus onset to indicate whether the scene contained a threat or a non-threat. After each response a short feedback video was presented indicating whether or not the participant responded correctly. Feedback was given for all four stimulus-response outcomes: hit, miss, false alarm, or correct rejection. If a threat was present and the participant reported a threat (a hit), the movie showed the scene progressing without harm and simultaneously a computer-generated voice-over complimented the participant for correct response. If a threat was present in the image but the participant reported a non-threat (a miss), the feedback movie showed the consequence of the failure to detect the threat (e.g. vehicle explosion, friendly casualty, building being destroyed) while playing a voice-over indicating that a threat had been missed. On a non-threat trial, if the participant responded that a threat was present (false alarm), the voice-over chastised the participant for the false alarm. Finally, if the participant correctly indicated that no threat was present on a non-threat trial (correct rejection), the voice-over praised the participant for correct response. None of these feedback videos provided specific information as to the identity of the threats, although they did allow participants to infer location and object type (e.g., bomb, sniper, or hidden gun). Training trials each lasted an average of 12 s. Each training block contained 60 trials, approximately half of which contained threats, and lasted 12 minutes. Participants completed four of these training blocks.

Test blocks were given before and after training and were similar to training blocks, except that no feedback was given after each response. Stimuli were presented for 2 s with a jittered ISI of 4–8 s. The ISI was a gray background with a crosshair fixation in the center of the screen. Participants had to respond within 3 s of stimulus onset or their response was not counted. Each test block included 50 stimuli, approximately half of which contained threats, and lasted 5 minutes.

### Transcranial Direct Current Stimulation Procedures

TDCS was applied using an ActivaDose II Iontophoresis Delivery Unit, which provides for delivery of a constant low level of direct current. Square-shaped (11 cm^2^) saline-soaked (0.9% sodium saline solution) sponge electrodes were attached to the participant with self-adhesive bandage strips. The anode was placed near electrode site F10 in the 10-10 EEG system, over the right sphenoid bone. The cathode was placed on the contralateral (left) upper arm. The site of the anode was selected based on our previous fMRI results showing that this brain region was the primary locus of neural activity associated with performance this task [23].

Participants in the active stimulation group received 2 mA current from the tDCS unit for a total of 30 minutes during the first two training blocks, beginning 5 minutes before the training started. Participants in the sham stimulation (control) group received 0.1 mA current over the same time period. The 0.1 mA current was used as a control condition, rather than the absence of stimulation, so as to equate aspects of the procedure (preparation and application of electrodes, attachment with adhesive strips, etc.). Another reason was to give the participant a degree of physical sensation that was somewhat similar to that of the 2 mA stimulation group while not reaching the level sufficient to affect brain function and behavior. Thus, the goal was to keep participants unaware as to which condition they were in, but we recognize that this represents only an approximation of a “single-blind” test procedure.

Participants first performed two pre-tDCS test blocks to determine baseline performance on the threat detection task (total duration about 10 minutes). After this, they performed two training blocks while receiving either active or sham tDCS stimulation (total duration about 25 minutes). Immediately after the completion of the second training block, the tDCS electrodes were removed and the participant continued on to complete two more training blocks without stimulation (total duration about 25 minutes). Thus, participants completed a total of six blocks of trials lasting a total of about 60 minutes in the baseline and learning phase of the experiment. To examine retention, we examined performance on an additional two test blocks, the first pair given immediately at the end of the first day of training (immediate retention condition), and the second pair given the next day (24-hour retention condition). Total participation time, including completing informed consent, entry questionnaires, participant instruction, task completion, and tDCS procedures, was about 1 hour 40 minutes on the first day and about 10 minutes on the second day.

### Sensation Questionnaire

A sensation questionnaire was administered at three different time points throughout the tDCS application. The first was given after the onset of the stimulation, the second after 5 minutes, and the third immediately after the first training block (approximately 17 minutes after the onset of stimulation). Participants were asked to rate their perceived sensations of itching, heat/burning, and tingling on a 10-point Likert scale; a response of 1 indicated that no sensation was being detected and 10 indicating extreme sensation. Stimulation was to be stopped immediately if participants reported a 7 or above on any of the sensation measures (This did not occur with any of the participants.)

### Data Analyses

The hit and false alarm rates for the learning phase of the threat detection task were computed for each of the six blocks of trials (two test, four training) for the active (2 mA) and sham stimulation (0.1 mA) groups. The hit and false alarm rates were then used to compute the parametric signal detection measures *d′* and *ß*. (The non-parametric signal detection measures *A′* and *C* were also computed and subjected to the same analyses, but are not reported here because the results were very similar to those for the *d′* and *ß* measures.) Each of the dependent measures was analyzed in 2 (group: active or sham)×6 (blocks) mixed analyses of variance (ANOVAs). All four performance measures were also computed for the immediate and 24-hour retention conditions (averaged over the two blocks in each condition) and subjected to 2 (group: active or sham)×3 (delay condition: baseline, immediate, or 24-hour retention) ANOVAs. The degrees of freedom for all F tests involving repeated measures factors were corrected for violations of the sphericity assumption by using the Greenhouse-Geisser procedure, and the alpha level was set at *p*<0.05.

## Results

### Learning

**Figure 2:**
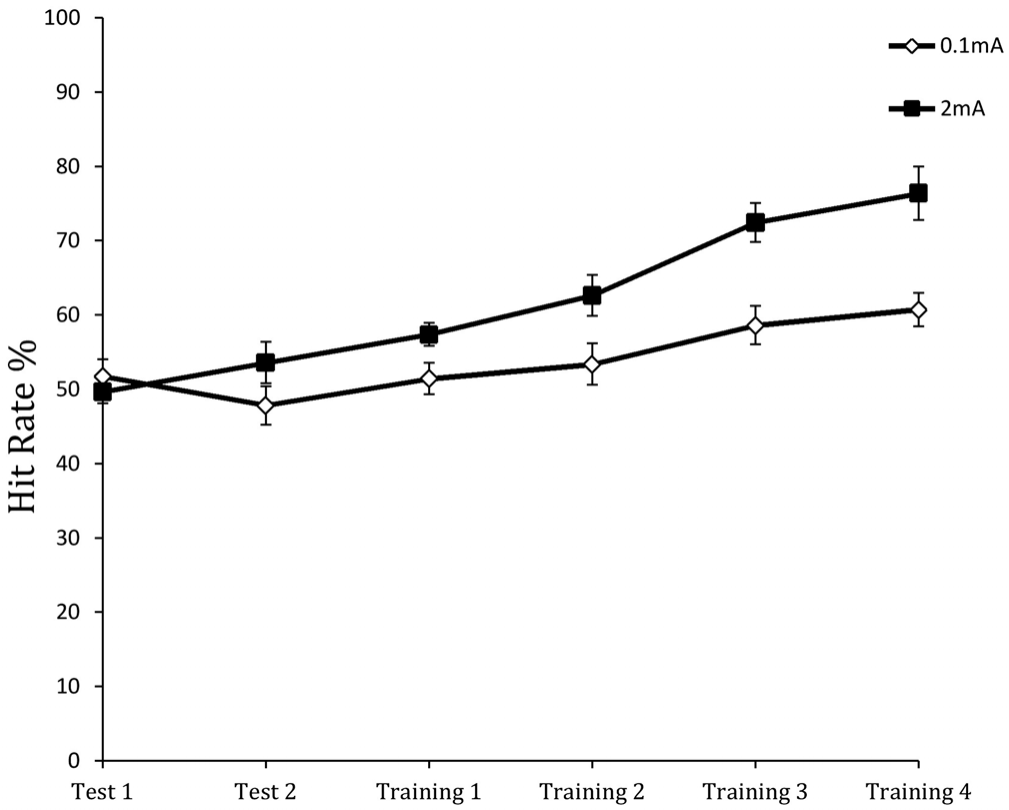
Mean percentage of correct responses on threat trials (hit rate) across the test and training blocks for the anodal (2 mA) and sham (0.1 mA) stimulation groups.

**Figure 3:**
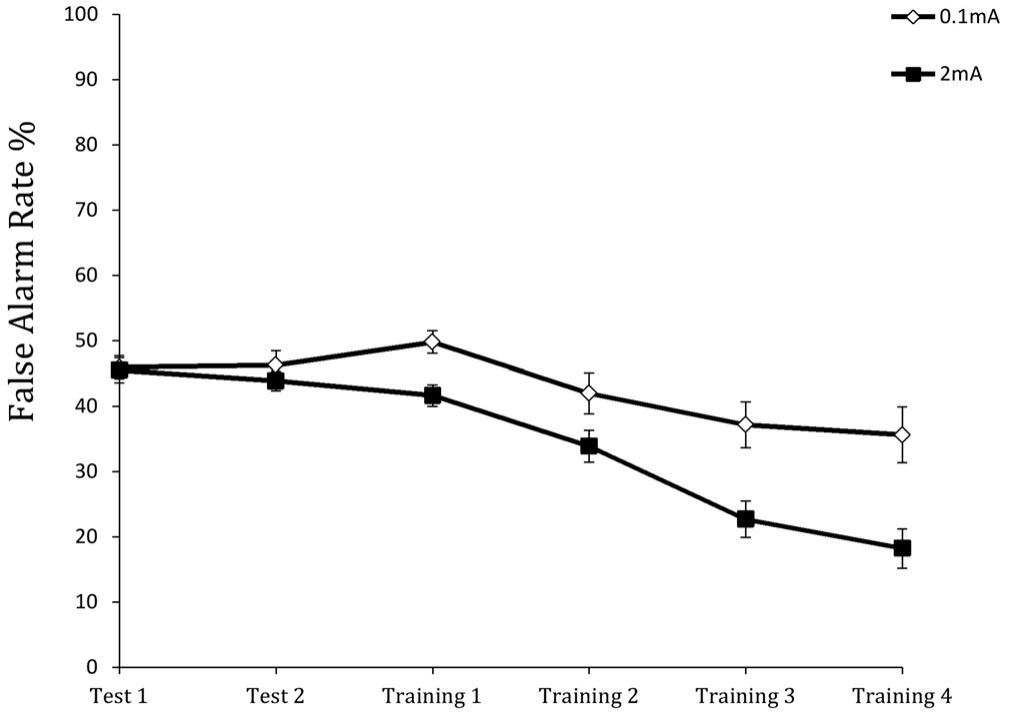
Mean percentage of falsely identified threats on non-threat trials (false alarm rate) across the test and training blocks for the anodal (2 mA) and sham (0.1 mA) stimulation groups.

Figures 2 and 3 show hit rate and false alarm rates for the six blocks of the learning phase of the study, including the first two baseline test blocks through the four training blocks. For hit rate, there was a significant effect of group, *F*(1,35) = 14.584, *p* = 0.001), blocks, *F*(3.179,111.279) = 23.139, *p*<0.0001, and the group×blocks interaction, *F*(3.179,111.279) = 4.109, *p*<0.01. As Figure 2 shows, the mean hit rate was about 50%—chance level performance—for both groups in the initial two baseline blocks, but the active stimulation (2 mA) group had significantly higher hit rates than the sham stimulation (0.1 mA) group in the subsequent training blocks, with the active stimulation group showing markedly better performance. Participants receiving active stimulation reached 76% hit rate at the end of training while the hit rate in the control (sham stimulation) group peaked at 61%.

For the false alarm rate, there were significant effects of group, *F*(1,35) = 7.050, *p*<0.05), blocks, *F*(2.577,90.180) = 34.854, *p*<0.001, and their interaction, *F*(2.577,90.180) = 5.314, *p*<0.01. As Figure 3 shows, the false alarm rate was about 50% for both groups in the initial baseline blocks, but declined thereafter during training. However, the active stimulation group showed a greater reduction, ending at 18% as opposed to 35% for the control group.

**Figure 4:**
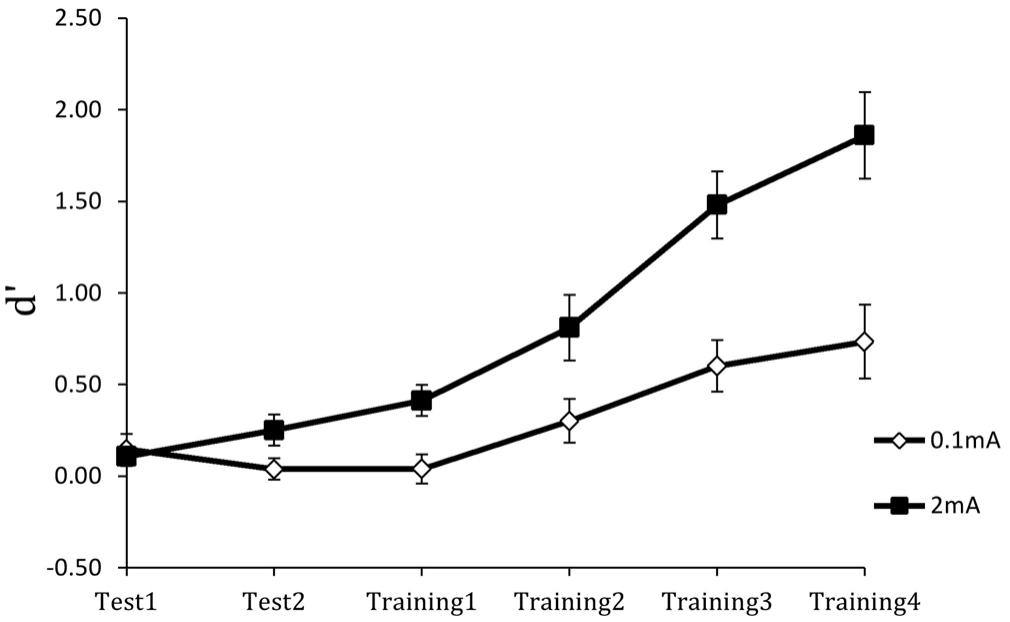
Mean perceptual sensitivity (*d′*) across the test and training blocks for the anodal (2 mA) and sham (0.1 mA) stimulation groups.

**Figure 5:**
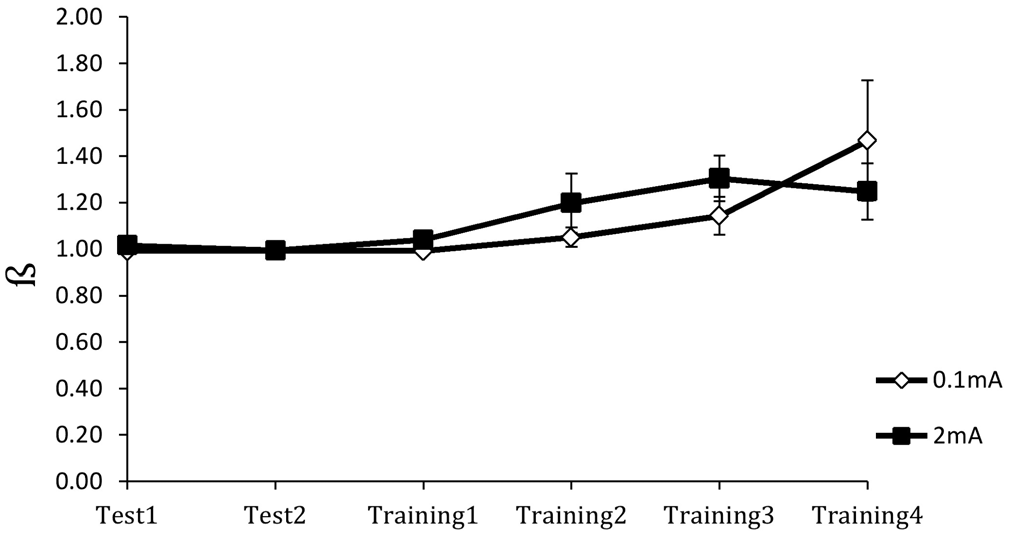
Mean response bias (*ß*) across the test and training blocks for the anodal (2 mA) and sham (0.1 mA) stimulation groups.

Figures 4 and 5 show the results during the learning phase for *d′* and *ß*. For *d′*, there were significant effects for group, *F*(1,35) = 12.676, *p*<0.001, blocks, *F*(2.126,74.414) = 45.392, *p*<0.001, and the group×blocks interaction, *F*(2.126,74.414) = 8.396, *p* = <0.001. As Figure 4 indicates, perceptual sensitivity was near zero in both groups during the baseline blocks but was significantly higher in the active stimulation group than in the control group during training. The significant group×blocks interaction shows that sensitivity increased more rapidly with training in the active stimulation group than in the control group. By the end of training, the 2 mA group had a *d′* of 1.86 while that for the 0.1 mA group was 0.73.

For the response bias measure *ß*, the main effect of group was not significant, *F*(1,35) = 0.133. The main effect of blocks was significant, *F*(1.738,60.844) = 5.121, *p*<0.05. The group×blocks interaction was not significant, *F*(1.738,60.844) = 1.116. As Figure 5 shows, there was a slight increase in *ß* over blocks towards the end of training in both groups—in a more conservative direction—but there were no differences between the active and sham stimulation groups in response bias.

### Retention

**Figure 6:**
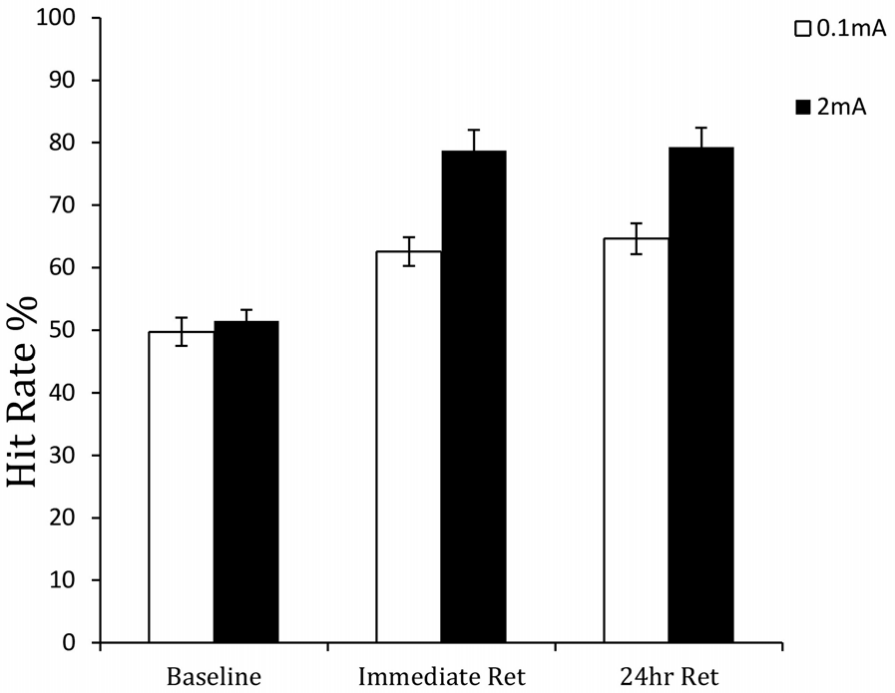
Mean hit rate in the pre-training baseline, immediate post-training retention test, and 24-hour retention test blocks for the anodal (2 mA) and sham (0.1 mA) stimulation groups.

For hit rate, the effect of group, *F*(1,35) = 15.537, *p*<0.001, delay condition, *F*(1.229,43.032) = 62.598, *p*<0.001, and the group×delay condition interaction, *F*(1.229,43.032) = 6.868, *p*<0.01, were significant. As Figure 6 shows, hit rates were higher in both retention periods than the baseline and were higher for the 2 mA group than for the 0.1 mA group. The 2 mA group improved their hit rate by 27.2% across the pre-training and immediate post-training test blocks. The hit rate remained at this relatively high level 24 hours later.

**Figure 7:**
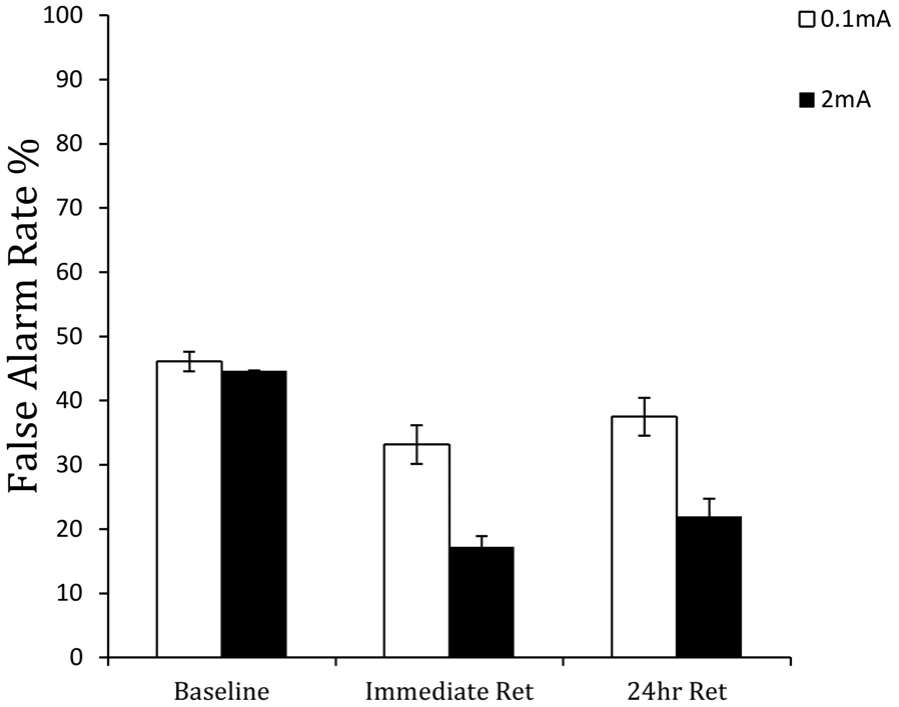
Mean false alarm rate in the pre-training baseline, immediate post-training retention test, and 24-hour retention test blocks for the anodal (2 mA) and sham (0.1 mA) stimulation groups.

For the false alarm rate, the effect of group, *F*(1,35) = 13.747, *p*<0.001, delay condition, *F*(1.689,59.116) = 89.787, *p*<0.001, and the group×delay condition interaction, *F*(1.689,59.116) = 13.412, *p*<0.001, were significant. Figure 7 shows that there was a reduction in false alarm rate by 27.4% in the 2 mA group from the pre-training to the immediate post-training test blocks. The false alarm rate remained at this level in the 24-hour retention test block.

**Figure 8:**
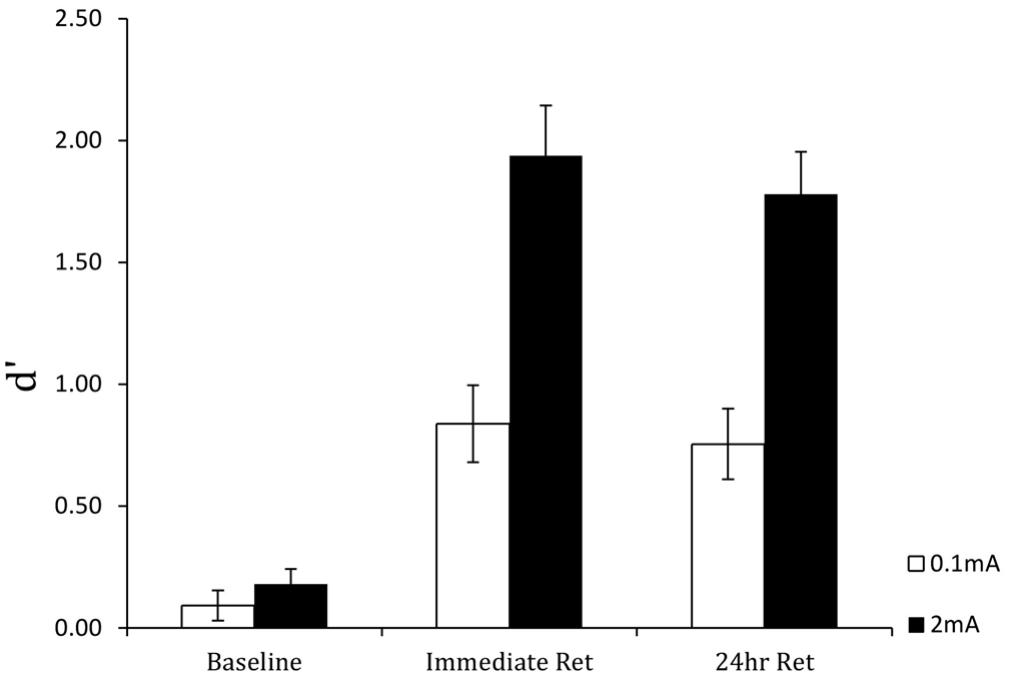
Mean perceptual sensitivity (*d′*) in the pre-training baseline, immediate post-training retention test, and 24-hour retention test blocks for the anodal (2 mA) and sham (0.1 mA) stimulation groups.

For *d′*, the effect of group, *F*(1,35) = 19.496, *p*<0.001, delay condition, *F*(1.307,45.738) = 84.335, *p*<0.001, and the group×delay condition interaction, *F*(1.307,45.738) = 14.065, *p*<0.001, were significant. As Figure 8 shows, *d*′ values were higher in both retention periods than the baseline and were higher for the 2 mA group than for the 0.1 mA group. There was a slight (∼8%) reduction in sensitivity in the 2 mA group from the immediate retention to the 24-hour retention periods.

Finally, for *ß*, the effect of group, *F*(1,35) = 0.040, delay condition, *F*(1.222,42.754) = 2.988, and the group×delay condition interaction, *F*(1.222,42.754) = .037, were all not significant. Thus, there were no significant effects of either training or retention on response bias.

### Sensation

The results of the sensation survey given at three time points throughout the stimulation procedure were averaged together for an overall sensation score for each sensation measure of tingling, heat, and itching (see Table 1). A significant difference in self-observed sensation scores between the 2 mA and 0.1 mA stimulation groups was found only for tingling, *F*(1,35) = 14.105, *p*<0.01, but not for heat *F*(1,35) = 1.084, nor itching, *F*(1,35) = 3.418. As Table 1 shows, however, the mean ratings for each sensation were near the bottom range of the 10-point scale, and the significant difference between groups for tingling was less than 1 point. To determine if learning was associated with perceived sensation, we conducted a correlational analysis for each sensation measure with the hit and false alarm rates of the two immediate post-training test blocks averaged together. Neither itching nor heat was significantly correlated with either the hit or false alarm rates (*r* values ranging from −.15 to .27). Tingling was moderately but significantly correlated with hit rate (*r* = .35, p = 0.03 uncorrected for multiple comparisons) but not with false alarm rate (*r* = −.18), *d′* (*r* = .24) or *ß* (*r* = .00).

**Table 1:** Mean sensation scores (on a 10-point scale, with 1 = no sensation and 10 = extreme sensation) for tingling, heat, and itching for the sham (0.1 mA) and anodal stimulation (2 mA) groups.

	Sham Stimulation (0.1 mA)	Anodal Stimulation (2 mA)
Itching	1.37	1.81
Heat	1.18	1.44
Tingling	1.57	2.51

## Discussion

This study examined whether stimulation of the right inferior frontal cortex using tDCS enhances learning and/or retention of a complex threat detection task, and if so, whether enhancement is based on increased perceptual sensitivity or an alteration in response bias. We found that, compared to a 0.1 mA sham stimulation control, stimulation with 2 mA tDCS increased perceptual sensitivity in detecting targets and accelerated learning in the task. The performance gain with tDCS was extensive: on completion of training, participants in the active stimulation group had more than double the perceptual sensitivity of the control group. Furthermore, the performance enhancement was maintained for 24 hours. Finally, the performance benefits associated with both skill acquisition and retention were rooted in an improvement in sensitivity (*d′*), rather than changes in response bias (*ß*).

Anodal 2 mA current was applied to the scalp electrode site F10 in the 10-10 EEG system. The resulting enhancement of performance in the threat detection task is consistent with our previous fMRI results [23] showing that the right inferior frontal cortex is a major locus of a distributed brain network that mediates performance on this task. The right parietal cortex is a part of this network and could also be a target for stimulation.

One possible explanation for the improvement in detection performance (hit rate) in the threat detection task is that tDCS increases general arousal, thereby leading to a change in response bias in the more liberal direction [25], which would increase the hit rate. However, computation of signal detection metrics showed that there were no significant effects of tDCS on the *ß* measure of response bias. Instead, the effect of brain stimulation was to enhance perceptual sensitivity, *d′*.

The improvement in perceptual sensitivity suggests that participants receiving tDCS were better able to encode stimulus features that distinguished targets and non-targets, which in turn led to accelerated learning and improved retention. Such effects are also consistent with the view that tDCS enhances attention, which is known to improve performance of perceptual detection tasks, particularly when targets are difficult to distinguish from non-targets [26]. In particular, attention has been found to improve the ability to detect concealed or obscured objects [27] and the intentional acts of other individuals [28], [29] in complex scenes. The mechanism by which attention enhances detection could be through the reduction of the influence of distracter objects that are close to the target [30], thereby enhancing detection of the target threat. This would suggest that stimulation-related enhancement of performance should be associated with increased activation of prefrontal cortical regions that provide top-down attentional control signals to inferior temporal cortical areas that mediate object recognition [31]. Our previous fMRI findings are consistent with this prediction [23].

In addition to examining whether tDCS enhances perceptual sensitivity during the acquisition of a threat detection task, the present study also investigated whether such performance enhancement can be retained over a period of 24 hours. The results were positive: 2 mA tDCS not only increased *d′* by more than a factor of two in the stimulation group compared to the control group, but this benefit was maintained when participants were tested without tDCS the next day.

There are a number of possible mechanisms underlying the retention of performance enhancement over a 24-hour period. First, anodal stimulation with tDCS may increase neuronal plasticity [32], [33], thereby enhancing the rate of learning compared to sham stimulation, and therefore also to retention of learning. A second possibility is the attentional explanation discussed previously with respect to the effects of tDCS on perceptual sensitivity. Attentional modulation with tDCS may increase effective perceptual acuity by allowing participants to detect the visual cues more easily, thereby improving encoding. This in turn may promote better retention, given that stimuli that are better attended and encoded are retained more effectively in memory [34]. However, we found no differences in the rate of forgetting over the 24-hour post-stimulation retention period between the anodal and sham stimulation groups, suggesting that once the threat stimuli were well encoded, performance showed the same (small) decay as in the untrained group. It is possible that differential retention rates could be observed over longer periods than that examined in this study, namely days or weeks. Retention of tDCS-based performance benefits over the long term is an important area for future research.

The results of the present study are encouraging with respect to translational applications. TDCS can be used to enhance sensitivity and accelerate learning of complex detection tasks in healthy individuals. Most previous tDCS studies have examined fairly simple perceptual and cognitive tasks having little ecological validity [17]. The use in the present study of tDCS training with a detection task that is more representative of real work environments is consistent with the goals of translational neuroscience [35] and with the neuroergonomic approach of applying neuroscience research to everyday and work settings [36], [37]. Other training techniques aimed at enhancing perceptual sensitivity in learning-impaired or autistic children have used psychophysical techniques such as slowing the rate at which stimuli are presented or increasing their contrast [38]. Training with tDCS can achieve the same goal as these other techniques but with the added advantage that training can be conducted with complex stimuli very similar to those encountered in real settings, thereby reducing concerns about transfer. More generally, tDCS also holds promise as a technique that could be used to remediate diminished perceptual sensitivity in these and other neurological and psychiatric disorders.
